# Evidence for Digital Mental Health Assessment Tools in the Post–COVID-19 Era: Protocol for a Systematic Review on Diagnostic Accuracy Across Age Groups

**DOI:** 10.2196/73492

**Published:** 2026-01-05

**Authors:** Kathryn Babbitt, Erin Lucy Funnell, Nayra Martin-Key, Eleanor Barker, Sabine Bahn

**Affiliations:** 1 Cambridge Centre for Neuropsychiatric Research, Department of Chemical Engineering and Biotechnology, University of Cambridge, Philippa Fawcett Drive, Cambridge, CB3 0AS, United Kingdom, 44 1223334151; 2 Engineering Library, Department of Engineering, University of Cambridge, Cambridge, United Kingdom

**Keywords:** systematic review, digital assessment tool, digital mental health, depression, generalized anxiety disorder, validity

## Abstract

**Background:**

Digital assessment tools in health care are increasingly used to aid clinicians in diagnosing mental health conditions. Particularly since the quarantine and isolation guidelines of the COVID-19 pandemic moved much of health care online, there has been an accelerated adoption of digital assessment tools. The diagnostic accuracy of digital mental health assessment tools for a range of psychiatric conditions has yet to be fully explored, especially for their use in populations of older adults and children.

**Objective:**

This systematic review aims to (1) summarize recent studies on digital self-report question-and-answer–based mental health assessment tools for use in all ages across a range of psychiatric conditions (eg, the type and number of questions, if available; reference tests; timing; and blinding procedures), (2) present their validity (ie, diagnostic accuracy), and (3) assess study quality and applicability.

**Methods:**

The PRISMA-P (Preferred Reporting Items for Systematic Review and Meta-Analysis Protocols) guided the development of this protocol. The protocol has been registered with PROSPERO. The searches were guided by the PICO (population, intervention, comparator, and outcome) framework. A systematic search was conducted of the following databases of literature published since 2021: MEDLINE, Embase, Cochrane Library, ASSIA, Web of Science Core Collection, CINAHL, and PsycINFO. Searches of clinical trial databases and hand searching of reference lists will be completed. Two authors have independently screened titles and abstracts of identified papers and selected studies according to eligibility criteria, resolving inconsistencies through discussion. Full texts were screened following the same process. The authors extracted data using the Covidence data extraction tool (Veritas Health Innovation Ltd; eg, sensitivity and specificity). Two authors will use the Quality Assessment of Diagnostic Accuracy Studies 2 (QUADAS-2) tool to assess risk of bias for each full-text inclusion.

**Results:**

Scoping for this review began in December 2024. Searches of databases were completed in January 2025. Full-text screening and identification of the relevant gray literature were completed by the end of August 2025, and the final review is expected to be completed by December 2025.

**Conclusions:**

The review aims to present the validity and quality of the diagnostic accuracy of digital mental health assessment tools across different ages (including children and older adults), particularly following the COVID-19 pandemic due to the exponential increase in development and use of such tools. This review will provide evidence for the wider deployment of digital mental health assessment tools across a wide age range. There will also be a discussion about future research for digital tools and avenues for policy around digital mental health assessments.

## Introduction

The implementation of digital technologies in health care has rapidly increased since the COVID-19 pandemic, as health care services were forced to operate in the unprecedented circumstances of quarantine. This posed great challenges as global health care systems had to adapt quickly to continue to serve patients, but there was also a unique opportunity for growth and improvements when systems were challenged to transition to online platforms [1,2]. Simultaneously, the surge in the global burden of mental health symptoms since the pandemic [3] resulted in an increased demand for evidence-based interventions that are accessible and can reduce the strain of mental health symptoms and conditions on the wider health care system. Currently, mental health care systems both in the United Kingdom and globally are struggling to address the clinical needs of patients, leaving help-seeking individuals on long waiting lists that span months and even years for certain diagnoses and presentations [4-6].

Digital mental health interventions pose a scalable solution to various system-level problems that may contribute to poor mental health care outcomes [7,8]. By their nature, digital tools are accessible and cost-effective, allowing anyone with access to a device and an internet connection to retrieve resources [9,10]. Individuals unable or less willing to seek in-person care now have the option to be seen online, potentially reducing stress associated with visits and the cost of travel [11]. Additionally, digital interventions can be easily deployed by health care professionals with adequate training [12], while also allowing for the results of the intervention to be accessed anywhere. One specific type of digital mental health intervention is assessments, which can range from computerized versions of traditional pen and paper assessments to sophisticated algorithms that incorporate artificial intelligence and machine learning to assess patients’ symptoms and clinical needs. Digital assessment tools vary greatly in their processes and may be used outside or alongside clinical care. Their outcomes vary as well, from providing diagnostic aid to serving as symptom severity checkers and risk assessments.

Digital assessment tools are part of an ever-changing landscape of health technologies, and while the evidence for the accuracy of digital assessment tools for mental health care has become more robust [13], continued updated research about the safety and validity of digital assessment tools is needed due to the potential for rapid development and deployment, which may result in the availability of low-quality tools [14]. It is also important to investigate the use and accuracy of digital assessment tools in the wider population, especially for younger and older people.

This study is intended to summarize recently published literature on digital assessment tools for mental health, with a particular focus on the years following the initial outbreak of the COVID-19 pandemic, which shifted many aspects of health care online. A previous systematic review from 2021 focused on digital mental health tools provided a basis to establish the quality of these assessments [13]. However, as outlined earlier, there is an opportunity for a more comprehensive summary for ages outside the range of 18 to 65 years, which was the population of interest in the previous review. As digital interventions become more widespread and more people rely on digital assessment tools, including young people [15] and older adults [16], it is important to understand the evidence for the validity of such tools for a wider age range, especially given the specific needs of these populations. Additionally, as the previous review ended in 2021, there is a need to examine literature in the years following the first wave of the pandemic. Summarizing the accuracy of these assessments in diverse age populations is imperative due to the exponential increase in their development and deployment since 2021 [17].

Therefore, the following research question is left unanswered in this literature: What is the evidence on the validity of digital assessment tools for mental health in children and young people (ie, under the age of 18 years, working-age adults (ie, between the ages of 18 and 65 years), and older adults (ie, aged >65 years) since the increased digitization of health care following the COVID-19 pandemic? In this study, we aim to present validity through evidence of diagnostic accuracy using typically reported metrics (ie, area under the curve [AUC], specificity, sensitivity, positive predictive value [PPV], negative predictive value [NPV], and accuracy). These metrics were chosen to be in line with STARD (Standards for Reporting of Diagnostic Accuracy Studies) 2015 guidelines, which suggest all diagnostic accuracy studies should mention a metric of accuracy such as sensitivity, specificity, predictive value (ie, PPV and NPV), or accuracy (ie, AUC) [18].

This systematic review aims to (1) summarize recent studies on digital self-report question-and-answer–based mental health assessment tools for use in all ages across a range of psychiatric conditions (eg, the type and number of questions [if available], reference tests, timing, and blinding procedures), (2) present their validity (ie, diagnostic accuracy), and (3) assess study quality and applicability. By presenting key evidence related to the diagnostic accuracy of digital mental health assessment tools, this review will inform our understanding of the quality and safety of currently available tools, as well as potentially support the wider acceptance and uptake of such tools.

## Methods

### Overview

The authors decided that the research questions would best be answered using a systematic review approach due to its applications for questions around international evidence, current practices, and intention to inform future research [19]. The primary objectives of this systematic review are to provide a comprehensive summary of existing digital mental health assessment tools and their validity in studies that use a gold standard reference. First, we will categorize the types of digital assessment tools available, distinguishing, for example, between digitized versions of traditional pen-and-paper psychiatric questionnaires and more advanced digital platforms such as those using machine learning approaches. Next, we will present the validity of these tools for each mental health condition under consideration. Finally, we will appraise the risk of bias and overall applicability of all included studies.

We have used the PRISMA-P (Preferred Reporting Items for Systematic Review and Meta-Analysis Protocols) [20] guidelines for the development of this protocol (Checklist 1). The protocol for this systematic review was registered with PROSPERO (CRD420250654734). The searches were guided by the PICO (population, intervention, comparator, and outcome) framework. For the full report, the PRISMA (Preferred Reporting Items for Systematic Reviews and Meta-Analyses) [21] will be used as a guide (Checklist 2). All documentation will be submitted on our adherence to all the above guidelines in the final report, and any changes to the protocol will be noted.

### Inclusion Criteria

#### Overview

To summarize the current evidence for the validity of digital mental health assessment tools, the inclusion and exclusion criteria were developed according to the PICO framework (Textbox 1). There is a rapidly growing number of digital assessment tools and substantial improvements in these technologies [22]. As digital tools in health care have been widely implemented, particularly since the pandemic [17], this review focuses on studies published since October 2021 to capture the current evidence base of the accuracy of these assessments across different age groups and to contribute to existing evidence for digital mental health assessment tools. October was chosen as the starting month to be included as it aligns with the end cutoff in a previous similar systematic review [13], therefore ensuring that the review builds on existing evidence and considers all published studies since then. Finally, studies were included regardless of study design.

Textbox 1. **PICO (population, intervention, comparator, and outcome) framework (see Multimedia Appendix 1 for full search strategies).**

**Population**

- Individuals assessed for symptoms of major depressive disorder, bipolar disorder, generalized anxiety disorder, panic disorder, social anxiety disorder, attention-deficit/hyperactivity disorder, autism spectrum disorders, insomnia, anorexia nervosa, bulimia nervosa, obsessive-compulsive disorder, psychosis, alcohol use disorder, substance use disorder, posttraumatic stress disorder, acute stress disorder, adjustment disorder, borderline personality disorder, emotionally unstable personality disorder, self-harm, and suicidality

**Intervention**

- Patient-completed digital question-and-answer–based mental health tools for assessment or similar assessments completed by a caregiver in the case of children or older adults

**Comparator**

- Reference standard including assessment by a clinician (eg, clinician interview), clinical diagnosis, self-reported diagnosis (including parent-reported diagnosis for children or young people, polysomnography for insomnia, or standardized structured or semistructured interview based on the *Diagnostic and Statistical Manual of Mental Disorders, Fifth Edition, Text Revision* [*DSM-5-TR*] and *International Classification of Diseases, 11th Revision* [*ICD-11*] criteria [or *DSM*-5 and *ICD-10* for older publications])

**Outcomes**

- Validity (eg, accuracy, area under the curve, sensitivity, specificity, positive predictive value, and negative predictive value)

#### Population

All study locations, mental health condition severity, and populations were included regardless of gender or ethnicity. While the previously referenced systematic review included working-age adults (aged 18-65 years) [13], the population of interest for this review has been expanded to include individuals of any age. The inclusion of a wider age range provides more evidence for the validity of digital mental health assessment tools in both children and older adults.

#### Intervention

Research that includes a digital question-and-answer–based mental health assessment tool intended for the assessment of any of the mental health conditions of interest specified in the population was included. The intervention required for inclusion in this review was defined as digital question-and-answer–based mental health tools for assessments that were completed by a patient or user. Because this review includes children and older adults, studies with tools aimed to investigate this population that require additional input from a parent or caregiver were included when applicable.

#### Comparator

To be included, studies must have had a “gold standard” comparator (ie, a clinician assessment, a structured clinical interview, self- or parent-reported diagnosis, or a clinical diagnosis). We also considered polysomnography to be a “gold standard” comparator for insomnia. Identification of this comparator was assessed via screening of the title, abstract, or full text, rather than defined through searches.

#### Outcome

The primary outcome measures of the study are metrics of validity and accuracy for question-and-answer–based digital assessment tools. This includes metrics such as AUC, specificity, sensitivity, PPV, and NPV.

### Exclusion Criteria

#### Overview

Exclusion criteria were developed to ensure the inclusion of the most relevant studies. To prevent including papers multiple times in the analysis, previous systematic reviews were excluded, although their reference lists were hand-searched for relevant papers for inclusion. While excluded, systematic reviews of similar topics were saved to compare the results of this review with previous results.

Other exclusion criteria were literature published before October 2021, papers with fewer than 500 words, ongoing clinical trials without results, papers published in non-English languages, editorials, opinion pieces, newspaper articles, and other various forms of popular media. Non–peer-reviewed literature (eg, dissertations and theses) was also excluded.

#### Population

Studies that specifically focus on military personnel and veterans, athletes, incarcerated or imprisoned individuals, detained individuals, police officers or other emergency personnel, or pregnant people were excluded. The only exceptions were studies that provide evidence for an assessment tool for post-traumatic stress disorder, where the experiences of these populations (eg, war, detention, and traumatic birth) may be relevant due to the diagnostic criteria. Studies that evaluate assessment tools designed for use in currently psychotic individuals, for assessment within the context of mental health crises or emergency mental health presentations within emergency department settings (eg, accident and emergency and emergency room), or use in groups solely due to a diagnosis of physical illnesses (eg, patients with cancer) were also excluded. Studies that consist of nonhuman studies were excluded.

#### Intervention

Studies with interventions that are designed to predict future mental illnesses and high-risk behaviors (eg, suicidality, suicide attempts, suicidal acts, and self-harm) were excluded. These tools were considered out of scope for this study because they fall outside the clinical use cases investigated in this review and have been identified as possibly high-risk for use in health care settings [23]. We aimed to look at tools that assess current mental health conditions rather than tools that predict the likelihood of future mental illnesses and high-risk behaviors. Interventions that are nondigital (ie, pen and paper) question-and-answer–based tools were excluded. Studies that evaluate digital tools that use other assessment methods outside of question-and-answer–based methods such as blood tests, imaging, genome analyses, wearables, speech biomarkers, and other digital biomarkers were excluded.

#### Comparator

Literature that does not address the outlined research question or include the specified reference standards was also excluded.

#### Outcomes

Studies that do not include one of the primary outcome measures of validity and accuracy for question-and-answer–based digital assessment tools were also excluded.

### Search Strategies

A medical librarian completed formal searches on January 14, 2025, of the following databases: MEDLINE (via Ovid), Embase (via Ovid), Cochrane Library, ASSIA (via ProQuest), Web of Science (Core Collection), CINAHL (via EBSCOhost), and PsycINFO (via EBSCOhost). Searches of the ClinicalTrials.gov database [24] were also completed by the medical librarian on January 28, 2025. Authors KB and EF completed searches of the World Health Organization clinical trial database [25] on February 10, 2025. To build on the existing literature [13] and to review digital tools for mental health assessment after the COVID-19 pandemic, searches were conducted for literature published in and after 2021. As this systematic review aims to provide a comprehensive view of the current state of digital assessment tools, the authors conducted additional gray literature searches. Gray literature searches through Google were completed on February 10, 2025, by authors KB and EF in collaboration with the medical librarian.

While previous systematic reviews without meta-analyses were excluded from this analysis, hand searches were performed of the reference lists of relevant systematic reviews captured by database searches to identify studies that were then screened against the eligibility criteria.

The included search terms were adapted from the previously published systematic review [13], which demonstrated an effective search strategy for identifying relevant studies. The full search strategy can be found here (Multimedia Appendix 1).

### Screening and Study Selection

Following the formal searches done by the medical librarian and gray literature searches, all of the identified literature was stored in Covidence (Veritas Health Innovation Ltd), a systematic review software. The literature was screened to remove duplicates before it was uploaded to Covidence.

Two independent reviewers (KB and EF) worked in Covidence to screen titles and abstracts against predetermined eligibility and exclusion criteria. Papers were flagged by the authors to include or exclude or to label if they were unsure. Any disagreements about whether to include or exclude papers were resolved by discussion. Following the initial screening, both reviewers screened the full texts of all included papers to determine their final eligibility based on the predetermined inclusion and exclusion criteria. Any disagreements were discussed, and when unable to come to a conclusion, a third reviewer (SB) was consulted.

Reasons for exclusion at the full-text screening stage were recorded in the Covidence software. The authors documented the number of papers included and excluded in every stage of review in the PRISMA 2020 flow diagram.

Results from gray literature searches in Google and the World Health Organization clinical trials database were screened against the same inclusion and exclusion criteria in a spreadsheet (Multimedia Appendix 2). Due to the comprehensive nature of the searches and the feasibility of screening such a large number of results, the decision was made not to conduct hand searching of references when systematic reviews were pulled from the gray literature searches.

### Data Extraction

Data are currently being extracted via the standardized Covidence data extraction form. Two reviewers (KB and ELF) will complete each form for every included paper. The following data are being extracted: general publication information (author, date, title, and location of study), study design, sample characteristics (size, mean age or age range, gender, and ethnicity breakdowns), mental health conditions addressed by the study, assessment type (including the index test and reference standard used in the study), and the relevant outcome measures addressed by the study (eg, PPV, NPV, AUC, sensitivity, specificity, and accuracy). Where reported, the authors will extract data on cutoff scores for the index tests and the performance at each cutoff. In the case that multiple cutoffs are presented, if there are no suggestions of the optimal cutoffs or suggested cutoffs based on previous literature in the paper, then no associated data will be extracted.

The reviewers will continue to compare data extraction forms for each study and resolve any discrepancies. Anything that remains unresolved will be decided by a third reviewer (SB). Following this, all extracted data will then be transferred to a shared online spreadsheet.

### Quality Appraisal

To evaluate the quality of the selected studies, the Revised Tool for the Quality Assessment of Diagnostic Accuracy Studies (QUADAS-2) is being used [26]. Two independent reviewers (KB and EF) are completing this assessment for all included studies using a shared online spreadsheet while blinded to the other reviewer’s responses. The QUADAS-2 is intended to evaluate possible biases and applicability of the study in relation to the research question for the review. The assessment consists of 4 domains, each of which addresses different aspects of the study. The domains are patient selection, index tests, reference standard, and flow and timing. Each domain consists of a subdomain about risk of bias, and three have a subdomain about applicability. The subdomains consist of questions that the rater evaluates as either “low,” “unclear,” or “high.” The subdomains are then evaluated together for the larger domain to reach a final decision about the risk of bias and concern about applicability. This method will be completed for all included studies. The results of the 2 reviewers will be compared. There will be a discussion about reaching a consensus when the reviewers have reached different conclusions, and a third reviewer will be consulted as needed (SB). A table will be created with the results.

### Data Synthesis

Due to the anticipated heterogeneity of studies, a descriptive approach will be used for results synthesis. A meta-analysis will not be pursued because of the potential range of findings, conditions included, and extended inclusion of all ages, and the potential for a large number of different screening tools to be pulled. Tables will also be used to present findings of extracted data (ie, study name, authors, year of publication, demographics, index test, reference standard, and outcome data of the index test’s performance). Results will be summarized based on the mental health condition and population (ie, children and young people, working-age adults, and older adults), and the current guidelines on the validity of digital tools will be described.

## Results

Scoping of this review began in December 2024. All database searches were completed on February 10, 2025. Manual searching of reference lists was completed on August 25, 2025, and the final review with analysis is expected to be completed by December 2025. The final results will be submitted to a peer-reviewed journal for dissemination.

Gray literature searches from Google included 55 URLs pulled from using an advanced search string (Multimedia Appendix 2).

**Figure 1.**
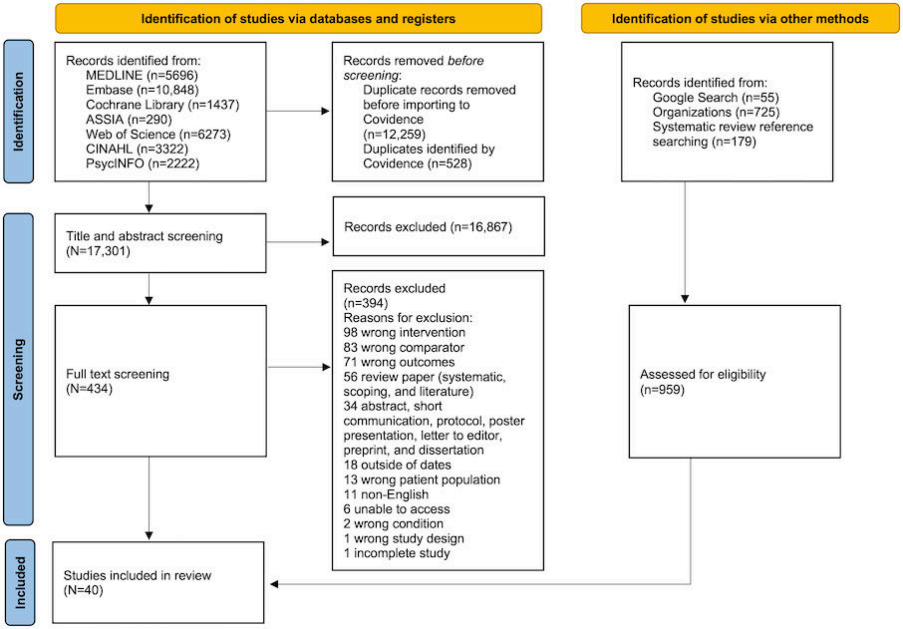
PRISMA (Preferred Reporting Items for Systematic Reviews and Meta-Analyses) 2020 flow diagram for study selection, which included searches of databases, registers, and other sources.

Currently, at the data extraction stage of the review, there are 40 studies that investigate 50 tools and screen for 17 conditions. The number of studies included at each stage is recorded in a PRISMA flowchart (Figure 1).

## Discussion

### Principal Findings

We hypothesize that, in line with previous systematic reviews [13], the evidence for the accuracy of digital assessment tools will be mixed. We anticipate that a large number of studies will fit the inclusion criteria due to the increased need for and implementation of digital interventions in the years after the COVID-19 pandemic. We also anticipate that proportionally, there will be fewer studies validating the digitalization of paper-and-pen questionnaires and a high number of studies that use machine learning techniques due to the rise in machine learning methods over the past 5 years [27].

### Comparison With Prior Work

Previous similar reviews on digital assessment tools have focused on adult populations only [13] or the landscape of tools for specific conditions [28,29]. This study aims to complement and further these previous reviews by searching for an expanded age range, including both older adults and children, as well as by including multiple conditions within one review.

### Strengths and Limitations

The main strengths of this study are the range of findings possible due to the breadth of conditions included and the expanded age range, which will allow for novel insights on the use of digital mental health assessment tools beyond just focusing on the use in the adult population. However, the broadened scope of this study limits the possible conclusions drawn due to likely heterogeneity in the pulled literature. Additionally, it is important to note that while this study addresses a range of conditions, it is not exhaustive, and there are several psychiatric conditions that are not examined. Finally, this review focuses only on question-and-answer–based assessments, which do not cover the recent technological advances in other forms of assessment.

### Future Directions

Future work should consider summarizing the landscape of digital mental health assessment tools for conditions not examined in this study that have seen a significant increase in the years following the COVID-19 pandemic and pandemic-related lockdowns, such as health anxiety, specific phobias, and specific addictions, especially those related to technology. We also did not examine other conditions that were present with physical and mental health symptoms (eg, irritable bowel syndrome and chronic fatigue).

Finally, future work should consider a broader search strategy to include other digital tools for mental health assessments, which are not question-and-answer–based such as digital biomarkers and wearable technologies that have also seen a rise in uptake in recent years [30]. Given their currently limited use in clinical settings, they were not included in this study, but this analysis could contribute to a more comprehensive understanding of the current landscape for digital assessments in the field beyond what will be presented in the review.

### Conclusions

This review aims to expand on previous literature reviews regarding digital mental health assessment tools, particularly in light of the increase in deployment of digital technologies in health care following the COVID-19 pandemic. By doing this, the current validity and quality of digital mental health assessments in adult populations will be presented while also expanding to include children and young people as well as older people to address this gap in the literature. This review will potentially provide evidence for the safe and effective deployment of digital assessment tools in a wider patient population and explore the potential opportunities for digital assessment tools to support safe and cost-effective health care delivery. There will also be a discussion about the implications and potential avenues for policy changes and future research directions.

## Supplementary material

10.2196/73492 Multimedia Appendix 1 Search strategies.

10.2196/73492 Multimedia Appendix 2 World Health Organization (WHO) clinical trials and advanced Google searches.

10.2196/73492 Checklist 1 PRISMA-P checklist.

10.2196/73492 Checklist 2 PRISMA checklist.

## Abbreviations

AUC: area under the curve
NPV: negative predictive value
PPV: positive predictive value
PRISMA: Preferred Reporting Items for Systematic Reviews and Meta-Analyses
PRISMA-P: Preferred Reporting Items for Systematic Review and Meta-Analysis Protocols
QUADAS-2: Quality Assessment of Diagnostic Accuracy Studies 2
STARD: Standards for Reporting of Diagnostic Accuracy Studies

## References

[1] Budd J, Miller BS, Manning EM (2020). Digital technologies in the public-health response to COVID-19. Nat Med.

[2] Getachew E, Adebeta T, Muzazu SGY (2023). Digital health in the era of COVID-19: reshaping the next generation of healthcare. Front Public Health.

[3] COVID-19 pandemic triggers 25% increase in prevalence of anxiety and depression worldwide. World Health Organization.

[4] Punton G, Dodd AL, McNeill A (2022). “You’re on the waiting list”: an interpretive phenomenological analysis of young adults’ experiences of waiting lists within mental health services in the UK. PLoS ONE.

[5] Thomas KA, Schroder AM, Rickwood DJ (2021). A systematic review of current approaches to managing demand and waitlists for mental health services. Mental Health Rev J.

[6] Edbrooke-Childs J, Deighton J (2020). Problem severity and waiting times for young people accessing mental health services. BJPsych Open.

[7] Spadaro B, Martin-Key NA, Funnell E, Benáček J, Bahn S (2023). Opportunities for the implementation of a digital mental health assessment tool in the United Kingdom: exploratory survey study. JMIR Form Res.

[8] Buck B, Kadakia A, Larsen A, Tauscher J, Guler J, Ben-Zeev D (2025). Digital interventions for people waitlisted for mental health services: a needs assessment and preference survey. Pract Innov (Wash D C).

[9] Gentili A, Failla G, Melnyk A (2022). The cost-effectiveness of digital health interventions: a systematic review of the literature. Front Public Health.

[10] Richards D, Enrique A, Eilert N (2020). A pragmatic randomized waitlist-controlled effectiveness and cost-effectiveness trial of digital interventions for depression and anxiety. NPJ Digit Med.

[11] Sweeney GM, Donovan CL, March S, Forbes Y (2019). Logging into therapy: adolescent perceptions of online therapies for mental health problems. Internet Interv.

[12] Wosny M, Strasser LM, Hastings J (2023). Experience of health care professionals using digital tools in the hospital: qualitative systematic review. JMIR Hum Factors.

[13] Martin-Key NA, Spadaro B, Funnell E (2022). The current state and validity of digital assessment tools for psychiatry: systematic review. JMIR Ment Health.

[14] Neary M, Schueller SM (2018). State of the field of mental health apps. Cogn Behav Pract.

[15] Boydell KM, Hodgins M, Pignatiello A, Teshima J, Edwards H, Willis D (2014). Using technology to deliver mental health services to children and youth: a scoping review. J Can Acad Child Adolesc Psychiatry.

[16] Harerimana B, Forchuk C, O’Regan T (2019). The use of technology for mental healthcare delivery among older adults with depressive symptoms: a systematic literature review. Int J Ment Health Nurs.

[17] Li J (2023). Digital technologies for mental health improvements in the COVID-19 pandemic: a scoping review. BMC Public Health.

[18] Cohen JF, Korevaar DA, Altman DG (2016). STARD 2015 guidelines for reporting diagnostic accuracy studies: explanation and elaboration. BMJ Open.

[19] Munn Z, Peters MDJ, Stern C, Tufanaru C, McArthur A, Aromataris E (2018). Systematic review or scoping review? Guidance for authors when choosing between a systematic or scoping review approach. BMC Med Res Methodol.

[20] Moher D, Shamseer L, Clarke M (2015). Preferred Reporting Items for Systematic Review and Meta-Analysis Protocols (PRISMA-P) 2015 statement. Syst Rev.

[21] Liberati A, Altman DG, Tetzlaff J (2009). The PRISMA statement for reporting systematic reviews and meta-analyses of studies that evaluate health care interventions: explanation and elaboration. PLoS Med.

[22] Chang JE, Lai AY, Gupta A, Nguyen AM, Berry CA, Shelley DR (2021). Rapid transition to telehealth and the digital divide: implications for primary care access and equity in a post-COVID era. Milbank Q.

[23] Alderman J, Riley R, Parekh D, Summers C, Liu X, Denniston A (2025). Hidden risks of predictive models in healthcare. BMJ Evid Based Med.

[24] ClinicalTrials.gov.

[25] International Clinical Trials Registry Platform. World Health Organization.

[26] Whiting PF, Rutjes AWS, Westwood ME (2011). QUADAS-2: a revised tool for the quality assessment of diagnostic accuracy studies. Ann Intern Med.

[27] Madububambachu U, Ukpebor A, Ihezue U (2024). Machine learning techniques to predict mental health diagnoses: a systematic literature review. Clin Pract Epidemiol Ment Health.

[28] De Angel V, Lewis S, White K (2022). Digital health tools for the passive monitoring of depression: a systematic review of methods. NPJ Digit Med.

[29] Plummer F, Manea L, Trepel D, McMillan D (2016). Screening for anxiety disorders with the GAD-7 and GAD-2: a systematic review and diagnostic metaanalysis. Gen Hosp Psychiatry.

[30] Şahin Tokatlıoğlu T, Oflaz F, Semiz B (2025). Wearable technologies and psychiatry: strengths, weaknesses, opportunities, and threats analysis. Cyprus J Med Sci.

