# Supplementary material for: Evidence for Digital Mental Health Assessment Tools in the Post–COVID-19 Era: Protocol for a Systematic Review on Diagnostic Accuracy Across Age Groups
Source: JMIR Res Protoc. 2026 Jan 5;15:e73492. doi: 10.2196/73492 (PMC12768427; doi:10.2196/73492)
Supplement: Multimedia Appendix 1 [file resprot-v15-e73492-s001.docx]

***Multimedia Appendix 1***

**Evidence for Digital Mental Health Assessment Tools: Protocol for a Systematic Review on Diagnostic Accuracy Across All Age Groups**

**Search strategies:**

**Ovid MEDLINE(R) and Epub Ahead of Print, In-Process, In-Data-Review & Other Non-Indexed Citations, Daily and Versions <1946 to January 10, 2025>**

1 ((assess* or diagnostic* or "mood diar*" or PHQ or "PHQ-9" or GAD or "GAD-7" or questionnaire* or screen* or tool* or test* or "computerised adaptive test for mental health" or "computerized adaptive test for mental health" or "CAT-MH" or "e-PASS" or WSQ or TAPS or Nview or ADA or doctorlink or clinicom) adj5 (accura* or sensitiv* or specific* or "receiver operating characteristic*" or ROC or "area under the curve" or AUC or AUROC or "positive predictive value" or PPV or "negative predictive value" or NPV or precision or recall or "true positive rate" or TPR or "true negative rate" or TNR or valid* or "agreement rate")).ti,ab. 738856

2 exp "Surveys and Questionnaires"/ and exp "Sensitivity and Specificity"/ 72573

3 1 or 2 794173

4 (app or apps or application* or chatbot* or computer* or "conversational agent*" or device* or digital or "e-health" or ehealth or "e-mental health" or "emental health" or electronic or internet or mhealth or "m-health" or mobile* or online or phone* or smartphone* or "smart-phone*" or cellphone* or "cell-phone*" or telehealth or telemedicine or "text messag*" or web or software or algorithm* or tablet* or PC or PCs).ti,ab. 4228579

5 Mobile Applications/ or exp Computers/ or Telemedicine/ or exp Cell Phone/ or exp Internet/ 245692

6 4 or 5 4297807

7 ((mood or affective or anxiety or panic or eating or "obsessive compulsive" or "attention deficit hyperactivity" or stress or adjustment or personality) adj disorder*).ti,ab. 214766

8 (depress* or dysthymi* or "MDD" or bipolar or "social phobia*" or ADHD or autism or ASD or insomnia or "anorexia nervosa" or "bulimia nervosa" or OCD or schizophrenia or psychosis or PTSD or BPD or EUPD or "self harm" or "self-harm" or suicid*).ti,ab. 1044765

9 (mental* adj (health or ill* or disorder*)).ti,ab. 317884

10 ((alcohol* or drug* or substance*) adj (abuse or addict*)).ti,ab. 73182

11 mental health/ or exp mental disorders/ 1576011

12 7 or 8 or 9 or 10 or 11 2279427

13 3 and 6 and 12 14185

14 limit 13 to yr="2021 -Current" 5696

**Embase <1974 to 2025 January 09>**

1 ((assess* or diagnostic* or "mood diar*" or PHQ or "PHQ-9" or GAD or "GAD-7" or questionnaire* or screen* or tool* or test* or "computerised adaptive test for mental health" or "computerized adaptive test for mental health" or "CAT-MH" or "e-PASS" or WSQ or TAPS or Nview or ADA or doctorlink or clinicom) adj5 (accura* or sensitiv* or specific* or "receiver operating characteristic*" or ROC or "area under the curve" or AUC or AUROC or "positive predictive value" or PPV or "negative predictive value" or NPV or precision or recall or "true positive rate" or TPR or "true negative rate" or TNR or valid* or "agreement rate")).ti,ab. 1024914

2 exp questionnaire/ and (receiver operating characteristic/ or "sensitivity and specificity"/ or validity/ or accuracy/) 34924

3 1 or 2 1044154

4 (app or apps or application* or chatbot* or computer* or "conversational agent*" or device* or digital or "e-health" or ehealth or "e-mental health" or "emental health" or electronic or internet or mhealth or "m-health" or mobile* or online or phone* or smartphone* or "smart-phone*" or cellphone* or "cell-phone*" or telehealth or telemedicine or "text messag*" or web or software or algorithm* or tablet* or PC or PCs).ti,ab. 5231835

5 exp mobile application/ or exp computer/ or telemedicine/ or exp mobile phone/ or exp internet/ 409096

6 4 or 5 5335680

7 ((mood or affective or anxiety or panic or eating or "obsessive compulsive" or "attention deficit hyperactivity" or stress or adjustment or personality) adj disorder*).ti,ab. 285305

8 (depress* or dysthymi* or "MDD" or bipolar or "social phobia*" or ADHD or autism or ASD or insomnia or "anorexia nervosa" or "bulimia nervosa" or OCD or schizophrenia or psychosis or PTSD or BPD or EUPD or "self harm" or "self-harm" or suicid*).ti,ab. 1400937

9 (mental* adj (health or ill* or disorder*)).ti,ab. 395112

10 ((alcohol* or drug* or substance*) adj (abuse or addict*)).ti,ab. 104074

11 exp mental health/ or exp mental disease/ 3053532

12 7 or 8 or 9 or 10 or 11 3572784

13 3 and 6 and 12 26882

14 limit 13 to yr="2021 -Current" 10848

**Cochrane Library**

**Search Name:**

**Date Run: 13/01/2025 14:48:07**

**Comment:**

ID Search Hits

#1 (((assess* or diagnostic* or "mood diar*" or PHQ or "PHQ-9" or GAD or "GAD-7" or questionnaire* or screen* or tool* or test* or "computerised adaptive test for mental health" or "computerized adaptive test for mental health" or "CAT-MH" or "e-PASS" or WSQ or TAPS or Nview or ADA or doctorlink or clinicom) Near/5 (accura* or sensitiv* or specific* or "receiver operating characteristic*" or ROC or "area under the curve" or AUC or AUROC or "positive predictive value" or PPV or "negative predictive value" or NPV or precision or recall or "true positive rate" or TPR or "true negative rate" or TNR or valid* or "agreement rate"))):ti,ab,kw (Word variations have been searched) 70493

#2 MeSH descriptor: [Surveys and Questionnaires] explode all trees 79523

#3 MeSH descriptor: [Sensitivity and Specificity] explode all trees 22022

#4 #2 and #3 3708

#5 #1 or #4 73159

#6 (app or apps or application* or chatbot* or computer* or "conversational agent*" or device* or digital or "e-health" or ehealth or "e-mental health" or "emental health" or electronic or internet or mhealth or "m-health" or mobile* or online or phone* or smartphone* or "smart-phone*" or cellphone* or "cell-phone*" or telehealth or telemedicine or "text messag*" or web or software or algorithm* or tablet* or PC or PCs):ti,ab,kw (Word variations have been searched) 434604

#7 MeSH descriptor: [Mobile Applications] this term only 2234

#8 MeSH descriptor: [Computers] explode all trees 3127

#9 MeSH descriptor: [Telemedicine] this term only 4355

#10 MeSH descriptor: [Cell Phone] explode all trees 3704

#11 MeSH descriptor: [Internet] explode all trees 6840

#12 #6 or #7 or #8 or #9 or #10 or #11 434898

#13 (((mood or affective or anxiety or panic or eating or "obsessive compulsive" or "attention deficit hyperactivity" or stress or adjustment or personality) NEXT disorder*)):ti,ab,kw (Word variations have been searched) 45743

#14 ((mental* NEXT (health or ill* or disorder*))):ti,ab,kw (Word variations have been searched) 48720

#15 (((alcohol* or drug* or substance*) NEXT (abuse or addict*))):ti,ab,kw (Word variations have been searched) 10991

#16 MeSH descriptor: [Mental Health] this term only 3462

#17 MeSH descriptor: [Mental Disorders] explode all trees 110307

#18 ((depress* or dysthymi* or "MDD" or bipolar or "social phobia*" or ADHD or autism or ASD or insomnia or "anorexia nervosa" or "bulimia nervosa" or OCD or schizophrenia or psychosis or PTSD or BPD or EUPD or "self harm" or "self-harm" or suicid*)):ti,ab,kw (Word variations have been searched) 181180

#19 #13 or #14 or #15 or #16 or #17 or #18 262444

#20 #5 and #12 and #19 4005

Results were then limited to 2021 onwards.

***ASSIA via ProQuest*** *(completed January 14^th^, 2025)*

1. ti(((assess* or diagnostic* or "mood diar*" or PHQ or "PHQ-9" or GAD or "GAD-7" or questionnaire* or screen* or tool* or test* or "computeri?ed adaptive test for mental health" or "CAT-MH" or "e-PASS” or WSQ or TAPS or Nview or ada or doctorlink or clinicom) NEAR/5 (accura* or sensitiv* or specific* or "receiver operating characteristic*" or ROC or "area under the curve" or AUC or AUROC or "positive predictive value" or PPV or "negative predictive value" or NPV or precision or recall or "true positive rate" or TPR or "true negative rate" or TNR or valid* or "agreement rate"))) OR ab(((assess* or diagnostic* or "mood diar*" or PHQ or "PHQ-9" or GAD or "GAD-7" or questionnaire* or screen* or tool* or test* or "computeri?ed adaptive test for mental health" or "CAT-MH" or "e-PASS” or WSQ or TAPS or Nview or ada or doctorlink or clinicom) NEAR/5 (accura* or sensitiv* or specific* or "receiver operating characteristic*" or ROC or "area under the curve" or AUC or AUROC or "positive predictive value" or PPV or "negative predictive value" or NPV or precision or recall or "true positive rate" or TPR or "true negative rate" or TNR or valid* or "agreement rate")))
2. (MAINSUBJECT.EXACT("Questionnaires") OR MAINSUBJECT.EXACT("Psychological tests") OR MAINSUBJECT.EXACT("Psychiatric tests")) AND MAINSUBJECT.EXACT("Receiver operating characteristic analysis")
3. 1 or 2
4. ti((app or apps or application* or chatbot* or computer* or "conversational agent*" or device* or digital or "e-health" or ehealth or "e-mental health" or "emental health" or electronic or internet or mhealth or "m-health" or mobile* or online or phone* or smartphone* or “smart-phone*” or cellphone* or “cell-phone*” or telehealth or telemedicine or "text messag*" or web or software or algorithm* or tablet* or PC or PCs)) OR ab((app or apps or application* or chatbot* or computer* or "conversational agent*" or device* or digital or "e-health" or ehealth or "e-mental health" or "emental health" or electronic or internet or mhealth or "m-health" or mobile* or online or phone* or smartphone* or “smart-phone*” or cellphone* or “cell-phone*” or telehealth or telemedicine or "text messag*" or web or software or algorithm*or tablet* or PC or PCs))
5. MAINSUBJECT.EXACT("Laptop computers") OR MAINSUBJECT.EXACT("Handheld computers") OR MAINSUBJECT.EXACT.EXPLODE("Notebook computers") OR MAINSUBJECT.EXACT("Computers") OR MAINSUBJECT.EXACT.EXPLODE("Telemedicine") OR MAINSUBJECT.EXACT("Mobile phones") OR MAINSUBJECT.EXACT("Internet")
6. 4 or 5
7. ti(((mood or affective or anxiety or panic or eating or "obsessive compulsive" or "attention deficit/hyperactivity" or stress or adjustment or personality) NEAR/1 disorder*)) OR ab(((mood or affective or anxiety or panic or eating or "obsessive compulsive" or "attention deficit/hyperactivity" or stress or adjustment or personality) NEAR/1 disorder*))
8. ti((Depress* or dysthymi* or "MDD" or bipolar or "social phobia*" or ADHD or autism or ASD or insomnia or "anorexia nervosa" or "bulimia nervosa" or OCD or schizophrenia or psychosis or PTSD or BPD or EUPD or "self harm" or "self-harm" or suicid*)) OR ab((Depress* or dysthymi* or "MDD" or bipolar or "social phobia*" or ADHD or autism or ASD or insomnia or "anorexia nervosa" or "bulimia nervosa" or OCD or schizophrenia or psychosis or PTSD or BPD or EUPD or "self harm" or "self-harm" or suicid*))
9. ti((mental* NEAR/1 (health or ill* or disorder*))) OR ab((mental* NEAR/1 (health or ill* or disorder*)))
10. ti(((alcohol* or drug* or substance*) NEAR/1 (abuse or addict*))) OR ab(((alcohol* or drug* or substance*) NEAR/1 (abuse or addict*)))
11. MAINSUBJECT.EXACT("Mental health") OR MAINSUBJECT.EXACT.EXPLODE("Mental disorders")
12. 7 or 8 or 9 or 10 or 11
13. 3 and 6 and 12
14. limit 13 to yr="2021-Current"

**# Web of Science Search Strategy (v0.1)**

**# Database: Web of Science Core Collection**

**# Entitlements:**

**- WOS.IC: 1993 to 2025**

**- WOS.CCR: 1985 to 2025**

**- WOS.SCI: 1900 to 2025**

**- WOS.AHCI: 1975 to 2025**

**- WOS.BHCI: 2008 to 2025**

**- WOS.BSCI: 2008 to 2025**

**- WOS.ESCI: 2020 to 2025**

**- WOS.ISTP: 1990 to 2025**

**- WOS.SSCI: 1956 to 2025**

**- WOS.ISSHP: 1990 to 2025**

**# Searches:**

1: TS=(((Assess* or diagnostic* or "mood diar*" or PHQ or "PHQ-9" or GAD or "GAD-7" or questionnaire* or screen* or tool* or test* or "computeri?ed adaptive test for mental health" or "CAT-MH" or "e-PASS” or WSQ or TAPS or Nview or ada or doctorlink or clinicom) NEAR/5 (accura* or sensitiv* or specific* or "receiver operating characteristic*" or ROC or "area under the curve" or AUC or AUROC or "positive predictive value" or PPV or "negative predictive value" or NPV or precision or recall or "true positive rate" or TPR or "true negative rate" or TNR or valid* or “agreement rate”))) Date Run: Tue Jan 14 2025 09:14:02 GMT+0000 (Greenwich Mean Time) Results: 1211562

2: TS=(App or apps or application* or chatbot* or computer* or "conversational agent*" or device* or digital or "e-health" or ehealth or "e-mental health" or "emental health" or electronic or internet or mhealth or "m-health" or mobile* or online or phone* or smartphone* or “smart-phone*” or cellphone* or “cell-phone*” or telehealth or telemedicine or "text messag*" or web or software or algorithm* or tablet* or PC or PCs) Date Run: Tue Jan 14 2025 09:14:37 GMT+0000 (Greenwich Mean Time) Results: 14054037

3: TS=(((mood or affective or anxiety or panic or eating or "obsessive compulsive" or "attention deficit/hyperactivity" or stress or adjustment or personality) NEAR/0 disorder*)) Date Run: Tue Jan 14 2025 09:15:11 GMT+0000 (Greenwich Mean Time) Results: 328658

4: TS=((depress* or dysthymi* or "MDD" or bipolar or "social phobia*" or ADHD or autism or ASD or insomnia or "anorexia nervosa" or "bulimia nervosa" or OCD or schizophrenia or psychosis or PTSD or BPD or EUPD or "self harm" or "self-harm" or suicid*)) Date Run: Tue Jan 14 2025 09:15:38 GMT+0000 (Greenwich Mean Time) Results: 1620826

5: TS=((mental* NEAR/0 (health or ill* or disorder*))) Date Run: Tue Jan 14 2025 09:15:59 GMT+0000 (Greenwich Mean Time) Results: 477576

6: TS=(((alcohol* or drug* or substance*) NEAR/0 (abuse or addict*))) Date Run: Tue Jan 14 2025 09:16:18 GMT+0000 (Greenwich Mean Time) Results: 100375

7: #6 OR #5 OR #4 OR #3 Date Run: Tue Jan 14 2025 09:16:33 GMT+0000 (Greenwich Mean Time) Results: 2037500

8: #7 AND #2 AND #1 Date Run: Tue Jan 14 2025 09:16:45 GMT+0000 (Greenwich Mean Time) Results: 13772

9: #7 AND #2 AND #1 and 2021 or 2022 or 2023 or 2024 or 2025 (Publication Years) Date Run: Tue Jan 14 2025 09:16:55 GMT+0000 (Greenwich Mean Time) Results: 6273

***CINAHL via EBSCO*** *(completed 14^th^ January, 2025)*

1. TI ( ((assess* or diagnostic* or "mood diar*" or PHQ or "PHQ-9" or GAD or "GAD-7" or questionnaire* or screen* or tool* or test* or "computeri?ed adaptive test for mental health" or "CAT-MH" or "e-PASS" or WSQ or TAPS or Nview or ada or doctorlink or clinicom) N5 (accura* or sensitiv* or specific* or "receiver operating characteristic*" or ROC or "area under the curve" or AUC or AUROC or "positive predictive value" or PPV or "negative predictive value" or NPV or precision or recall or "true positive rate" or TPR or "true negative rate" or TNR or valid* or “agreement rate”)) ) OR AB ( ((assess* or diagnostic* or "mood diar*" or PHQ or "PHQ-9" or GAD or "GAD-7" or questionnaire* or screen* or tool* or test* or "computeri?ed adaptive test for mental health" or "CAT-MH" or "e-PASS” or WSQ or TAPS or Nview or ada or doctorlink or clinicom) N5 (accura* or sensitiv* or specific* or "receiver operating characteristic*" or ROC or "area under the curve" or AUC or AUROC or "positive predictive value" or PPV or "negative predictive value" or NPV or precision or recall or "true positive rate" or TPR or "true negative rate" or TNR or valid* or “agreement rate”)) )
2. ((MH "Surveys+") OR (MH "Questionnaires+")) AND (MH "Sensitivity and Specificity") OR (MH "ROC Curve")
3. 1 or 2
4. TI ( (app or apps or application* or chatbot* or computer* or "conversational agent*" or device* or digital or "e-health" or ehealth or "e-mental health" or "emental health" or electronic or internet or mhealth or "m-health" or mobile* or online or phone* or smartphone* or “smart-phone*” or cellphone* or “cell-phone*” or telehealth or telemedicine or "text messag*" or web or software or algorithm*) ) OR AB ( (app or apps or application* or chatbot* or computer* or "conversational agent*" or device* or digital or "e-health" or ehealth or "e-mental health" or "emental health" or electronic or internet or mhealth or "m-health" or mobile* or online or phone* or smartphone* or “smart-phone*” cellphone* or “cell-phone*”or telehealth or telemedicine or "text messag*" or web or software or algorithm*or tablet* or PC or PCs)
5. (MH "Mobile Applications") OR (MH "Computers and Computerization+") OR (MH "Telemedicine") OR (MH "Cellular Phone+")
6. 4 or 5
7. TI ( ((mood or affective or anxiety or panic or eating or "obsessive compulsive" or "attention deficit hyperactivity" or stress or adjustment or personality) N1 disorder*) ) OR AB ( ((mood or affective or anxiety or panic or eating or "obsessive compulsive" or "attention deficit hyperactivity" or stress or adjustment or personality) N1 disorder*) )
8. TI ( (depress* or dysthymi* or "MDD" or bipolar or "social phobia*" or ADHD or autism or ASD or insomnia or "anorexia nervosa" or "bulimia nervosa" or OCD or schizophrenia or psychosis or PTSD or BPD or EUPD or "self harm" or "self-harm" or suicid*) ) OR AB ( (depress* or dysthymi* or "MDD" or bipolar or "social phobia*" or ADHD or autism or ASD or insomnia or "anorexia nervosa" or "bulimia nervosa" or OCD or schizophrenia or psychosis or PTSD or BPD or EUPD or "self harm" or "self-harm" or suicid*) )
9. TI ( (mental* N1 (health or ill* or disorder*)) ) OR AB ( (mental* N1 (health or ill* or disorder*)) )
10. TI ( ((alcohol* or drug* or substance*) N1 (abuse or addict*)) ) OR AB ( ((alcohol* or drug* or substance*) N1 (abuse or addict*)) )
11. (MH "Mental Health") OR (MH "Mental Disorders+")
12. 7 or 8 or 9 or 10 or 11
13. 3 and 6 and 12
14. limit 13 to yr="2021 -Current"

***PsycINFO via EBSCO*** *(completed 14^th^ January, 2025)*

1. TI ( ((Assess* or diagnostic* or "mood diar*" or PHQ or "PHQ-9" or GAD or "GAD-7" or questionnaire* or screen* or tool* or test* or "computeri?ed adaptive test for mental health" or "CAT-MH" or "e-PASS” or WSQ or TAPS or Nview or ada or doctorlink or clinicom) N5 (accura* or sensitiv* or specific* or "receiver operating characteristic*" or ROC or "area under the curve" or AUC or AUROC or "positive predictive value" or PPV or "negative predictive value" or NPV or precision or recall or "true positive rate" or TPR or "true negative rate" or TNR or valid* or “agreement rate”)) ) OR AB ( ((Assess* or diagnostic* or "mood diar*" or PHQ or "PHQ-9" or GAD or "GAD-7" or questionnaire* or screen* or tool* or test* or "computeri?ed adaptive test for mental health" or "CAT-MH" or "e-PASS” or WSQ or TAPS or Nview or ada or doctorlink or clinicom) N5 (accura* or sensitiv* or specific* or "receiver operating characteristic*" or ROC or "area under the curve" or AUC or AUROC or "positive predictive value" or PPV or "negative predictive value" or NPV or precision or recall or "true positive rate" or TPR or "true negative rate" or TNR or valid* or “agreement rate”)) )
2. (DE "Surveys" OR DE "Online Surveys" OR DE "Questionnaires") AND (DE "Test Specificity" OR DE "Test Sensitivity")
3. 1 or 2
4. TI ( (App or apps or application* or chatbot* or computer* or "conversational agent*" or device* or digital or "e-health" or ehealth or "e-mental health" or "emental health" or electronic or internet or mhealth or "m-health" or mobile* or online or phone* or smartphone* or “smart-phone*” or cellphone* or “cell-phone*” or telehealth or telemedicine or "text messag*" or web or software or algorithm* or tablet* or PC or PCs) ) OR AB ( (App or apps or application* or chatbot* or computer* or "conversational agent*" or device* or digital or "e-health" or ehealth or "e-mental health" or "emental health" or electronic or internet or mhealth or "m-health" or mobile* or online or phone* or smartphone* or “smart-phone*” or cellphone* or “cell-phone*” or telehealth or telemedicine or "text messag*" or web or software or algorithm* or tablet* or PC or PCs) )
5. DE "Mobile Applications" OR DE "Computer Applications" OR DE "Mobile Phones" OR DE "Smartphones" OR DE "Computers" OR DE "Tablet Computers" OR DE "Telemedicine" OR DE "Mobile Health"
6. 4 or 5
7. TI ( ((mood or affective or anxiety or panic or eating or "obsessive compulsive" or "attention deficit/hyperactivity" or stress or adjustment or personality) N1 disorder*) ) OR AB ( ((mood or affective or anxiety or panic or eating or "obsessive compulsive" or "attention deficit/hyperactivity" or stress or adjustment or personality) N1 disorder*) )
8. TI ( (Depress* or dysthymi* or "MDD" or bipolar or "social phobia*" or ADHD or autism or ASD or insomnia or "anorexia nervosa" or "bulimia nervosa" or OCD or schizophrenia or psychosis or PTSD or BPD or EUPD or "self harm" or "self-harm" or suicid*) ) OR AB ( (Depress* or dysthymi* or "MDD" or bipolar or "social phobia*" or ADHD or autism or ASD or insomnia or "anorexia nervosa" or "bulimia nervosa" or OCD or schizophrenia or psychosis or PTSD or BPD or EUPD or "self harm" or "self-harm" or suicid*) )
9. TI ( (mental* N1 (health or ill* or disorder*)) ) OR AB ( (mental* N1 (health or ill* or disorder*)) )
10. TI ( ((alcohol* or drug* or substance*) N1 (abuse or addict*)) ) OR AB ( ((alcohol* or drug* or substance*) N1 (abuse or addict*)) )
11. DE "Mental Health" OR DE "Mental Disorders" OR DE "Affective Disorders" OR DE "Anxiety Disorders" OR DE "Autism Spectrum Disorders" OR DE "Bipolar Disorder" OR DE "Borderline States" DE "Eating Disorders" OR DE "Neurodevelopmental Disorders" OR DE "Personality Disorders" OR DE "Psychosis" OR DE "Serious Mental Illness" OR DE "Sleep Wake Disorders" OR DE "Stress and Trauma Related Disorders" OR DE "Substance Related and Addictive Disorders"
12. 7 or 8 or 9 or 10 or 11
13. 3 and 6 and 12
14. limit 13 to yr="2021-Current"

**Grey literature:**

***ClinicalTrials.gov*** *(completed January 28^th^, 2025)*

Condition: “mental health”; Other terms: “assessment tools”

***World Health Organization International Clinical Trials Registry Platform (ICTRP)*** *(completed February 10^th^, 2025)*

Search term: “mental health” AND “assessment”

***Advanced Google search*** *(completed February 10^th^, 2025)*

Search terms: "digital tools" AND "mental health" AND “assessment” AND “questionnaire” (site:.edu OR site:.ac OR site:.gov OR site:.org) Only the first 50 pages were searched.
